# Supplementary material for: Structures and transport dynamics of a Campylobacter jejuni multidrug efflux pump
Source: Nat Commun. 2017 Aug 1;8:171. doi: 10.1038/s41467-017-00217-z (PMC5537355; doi:10.1038/s41467-017-00217-z)
Supplement: Supplementary file 1 — Supplementary information [file 41467_2017_217_MOESM1_ESM.pdf]

Title of file for HTML: Supplementary Information
Description: Supplementary figures, supplementary tables, supplementary notes

Title of file for HTML: Peer Review File
Description:

SUPPLEMENTARY INFORMATION

a.

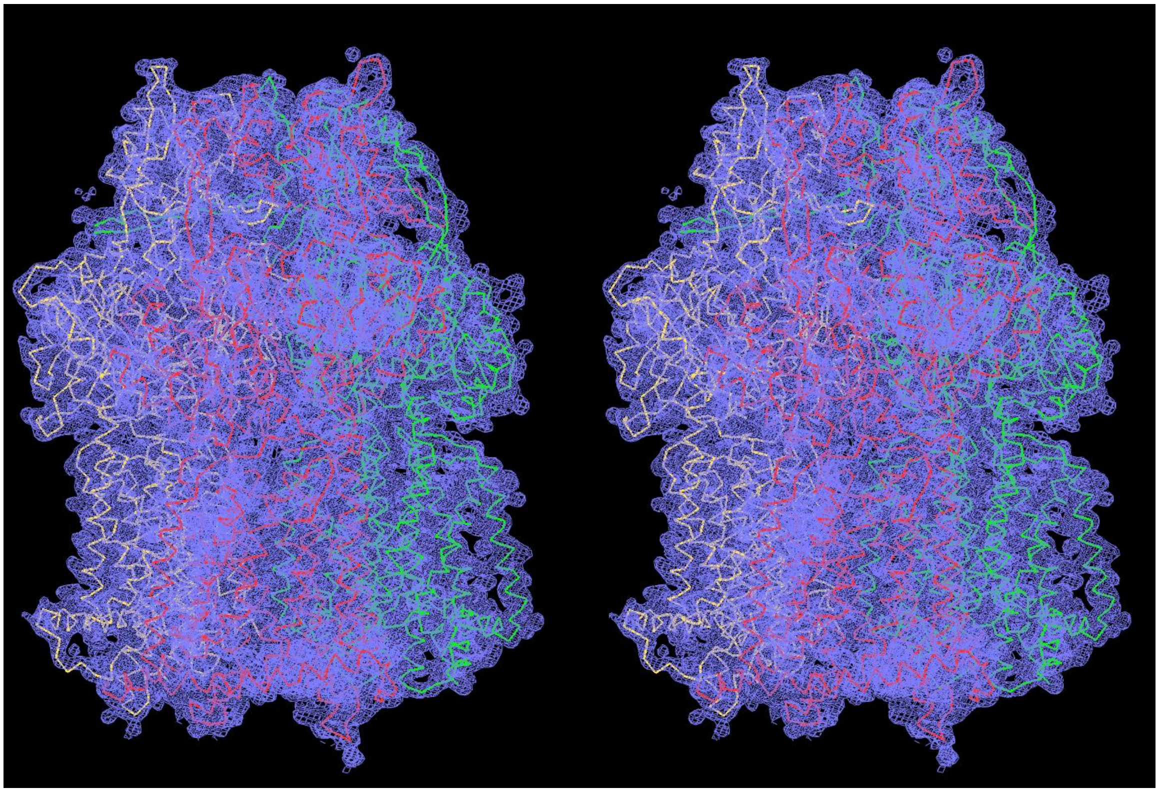

b.

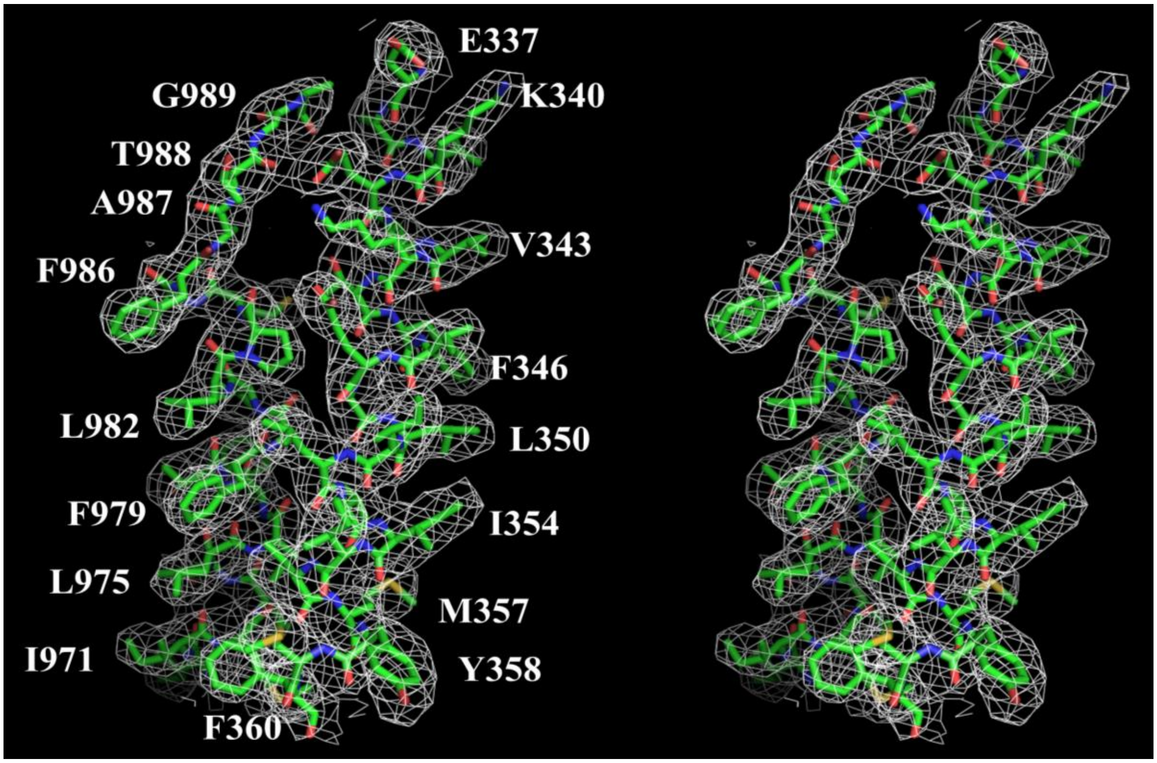

c.

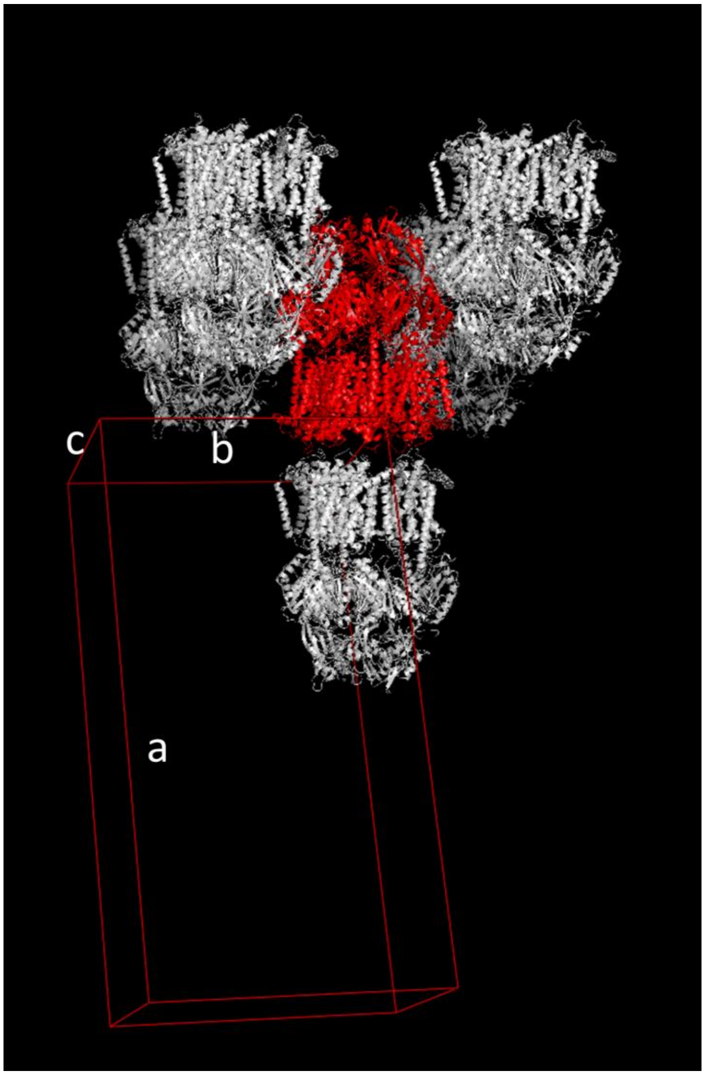

Supplementary Figure 1. Stereo view of the electron density maps of CmeB (form I) at a resolution of 3.15 Å. (a) The electron density maps are contoured at 1.2 σ. The Cα traces of the CmeB trimer in the asymmetric unit are included. (b) Representative section of the electron density in the vicinity of TMs 2 and 11 of CmeB. The electron density (colored white) is contoured at the 1.2 σ level and superimposed with the final refined model (green, carbon; red, oxygen; blue, nitrogen). (c) Packing diagram of the CmeB (form I) crystal structure.

a.

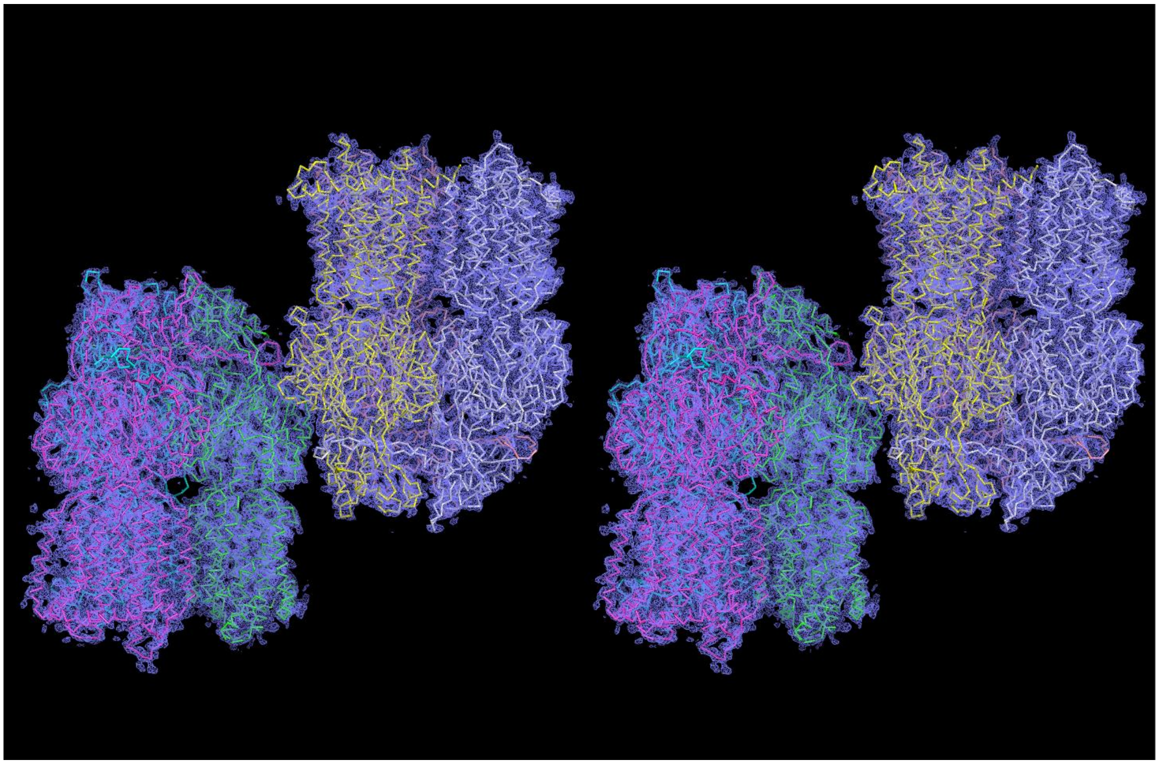

b.

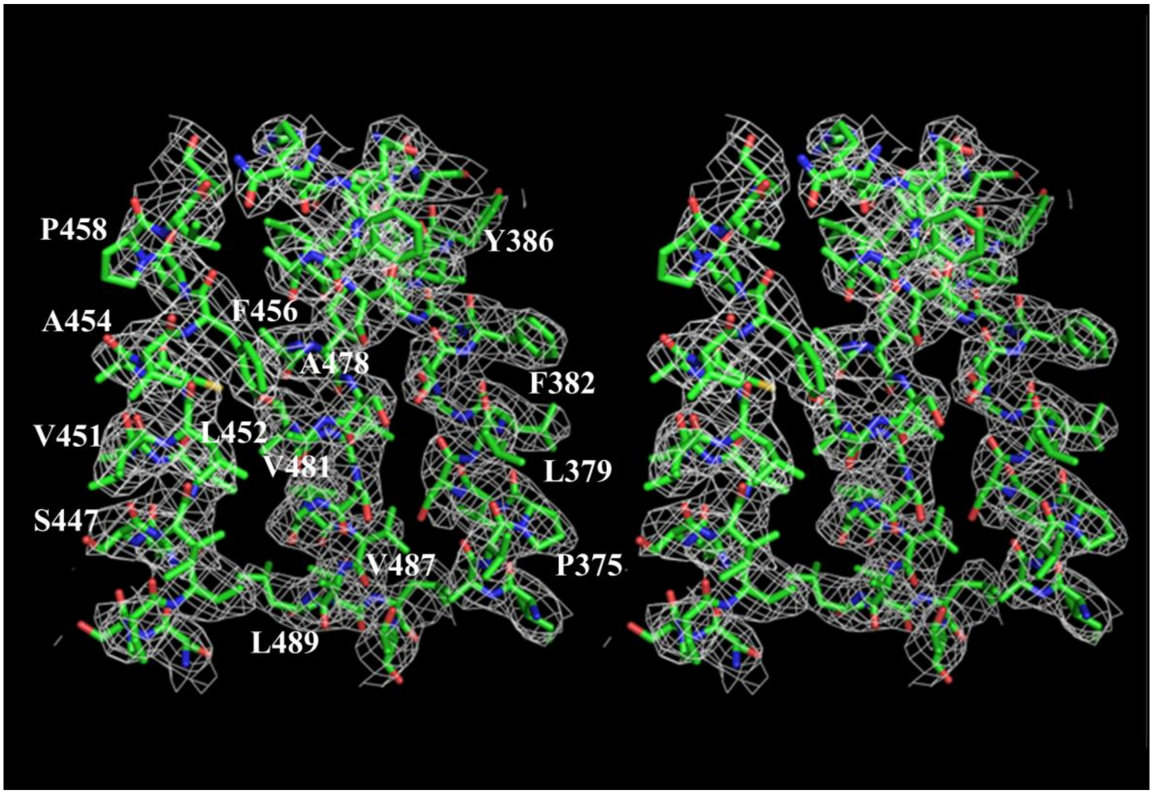
P458
Y386
A454
F456
A478
F382
V451
L452
V481
L379
S447
V487
P375
L489

c.

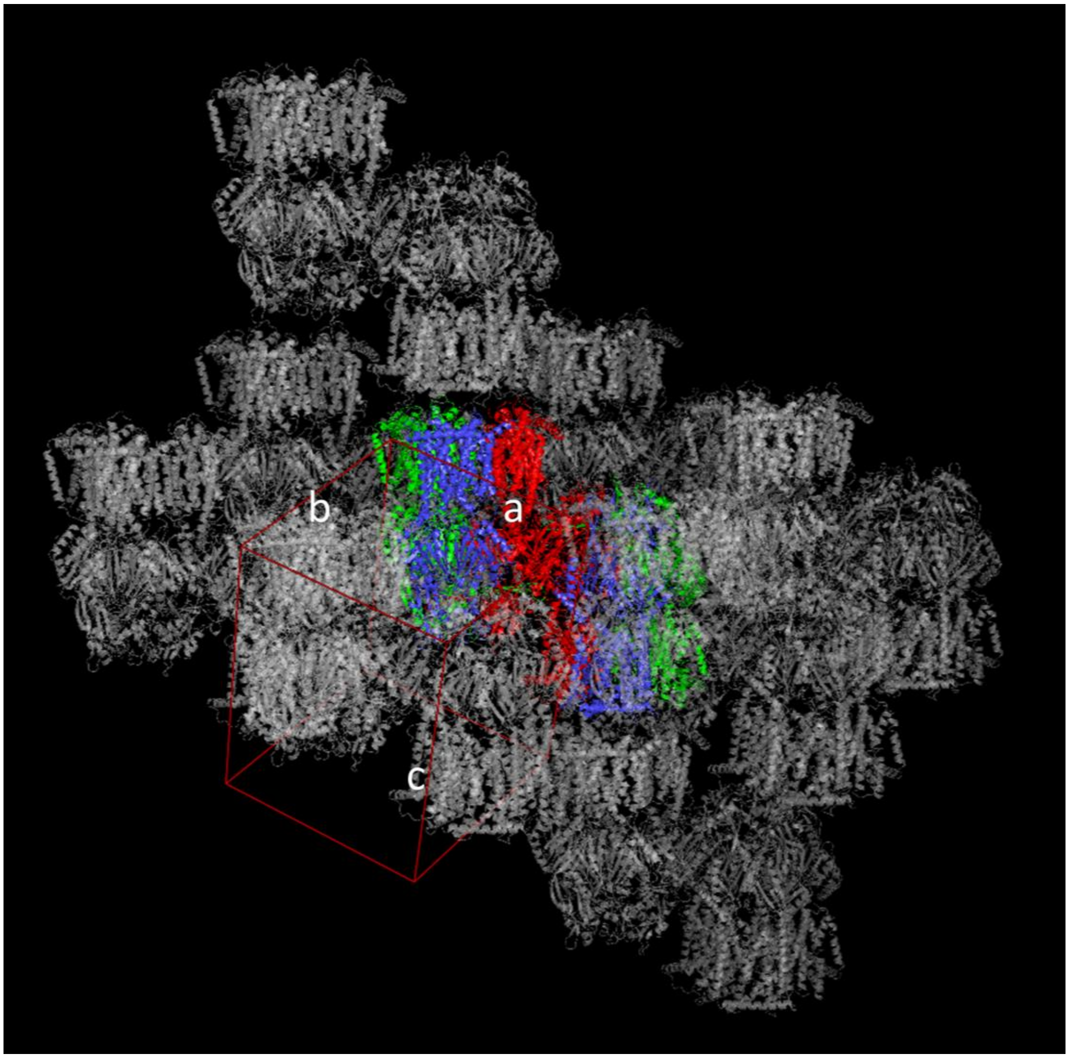

Supplementary Figure 2. Stereo view of the electron density maps of CmeB (form II) at a resolution of 3.55 Å. (a) The electron density maps are contoured at 1.2 σ. The Cα traces of the CmeB trimer in the asymmetric unit are included. (b) Representative section of the electron density in the vicinity of TMs 3, 5 and 6 of CmeB. The electron density (colored white) is contoured at the 1.2 σ level and superimposed with the final refined model (green, carbon; red, oxygen; blue, nitrogen). (c) Packing diagram of the CmeB (form II) crystal structure (red, extrusion protomer; blue, binding protomer; green, resting protomer).

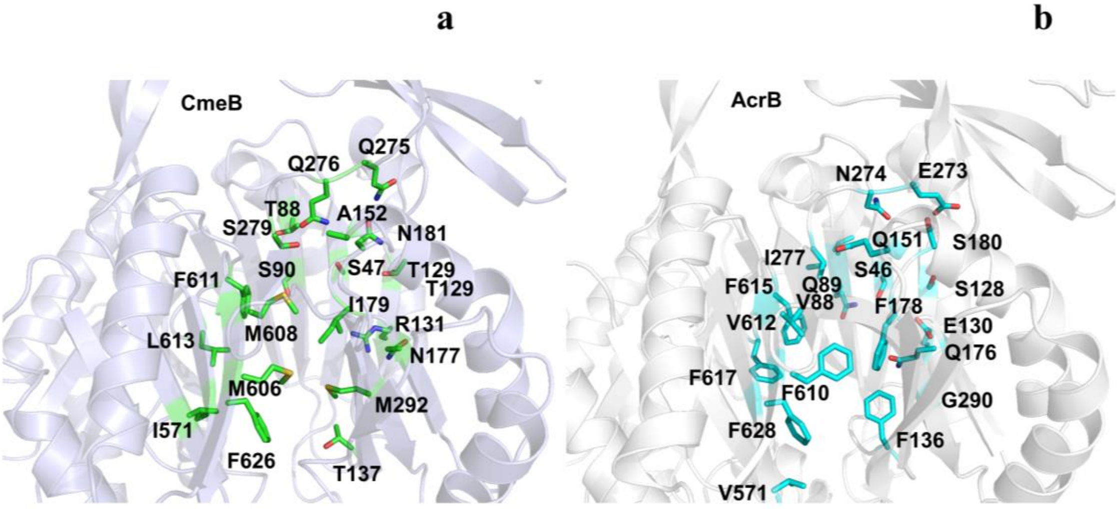

Supplementary Figure 3. Composition of the multidrug-binding pocket. (a) Residues located within the multidrug-binding site of the form II structure of CmeB are shown in green sticks. (b) The corresponding residues in the distal binding pocket of AcrB (PDB ID: 4dx5) are cyan sticks.

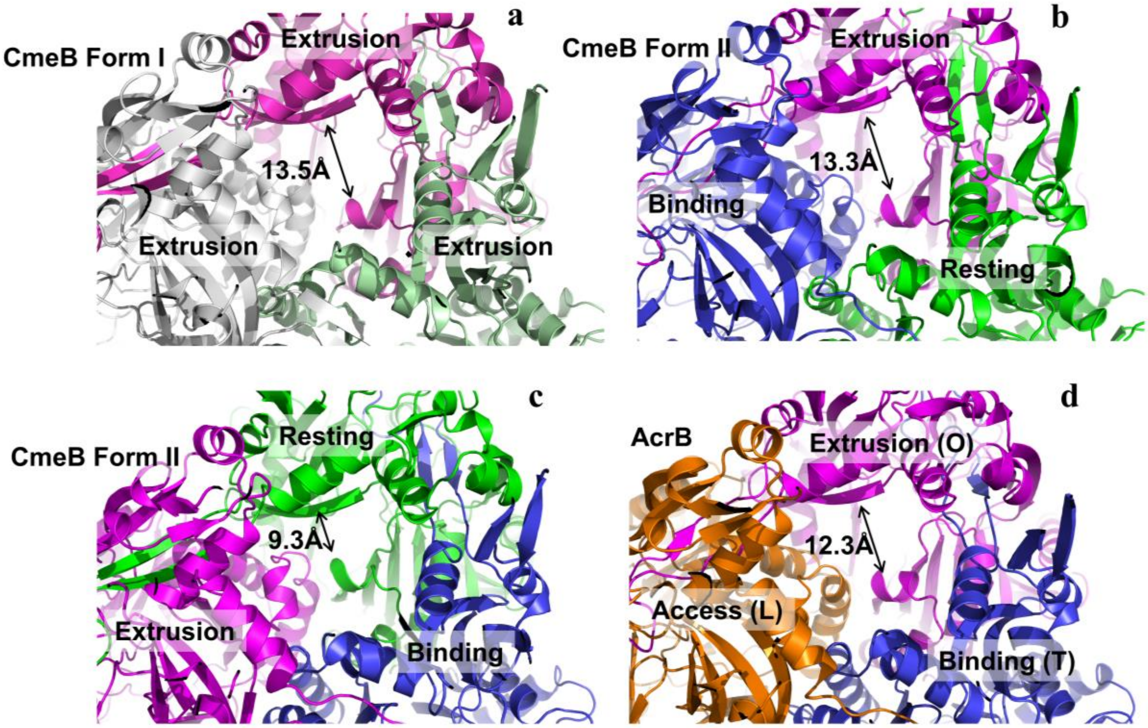

Supplementary Figure 4. The narrowest region of the periplasmic tunnels of CmeB and AcrB. The opening between residues L126 and Y752 of the (a) form I structure of the extrusion protomer of CmeB, (b) form II structure of the extrusion protomer of CmeB and (c) form II structure of the resting protomer of CmeB. (d) The corresponding opening between residues Q125 and Y758 of the extrusion protomer of AcrB. This opening is wide enough for drug molecules to pass through.

a b

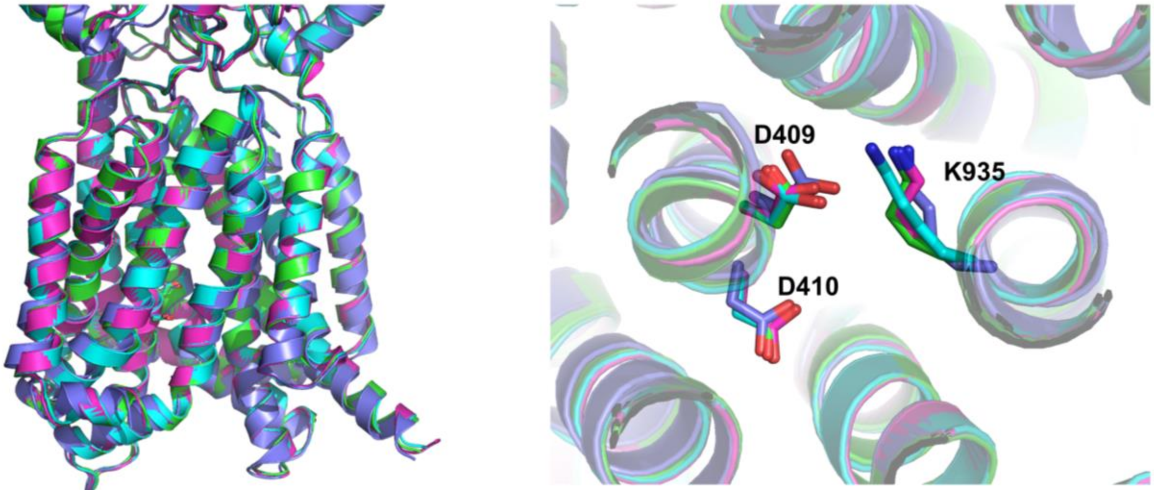

Supplementary Figure 5. Comparison of the structures of the extrusion protomers CmeB. (a) The conformation of the transmembrane helices of CmeB (green, form I protomer 1; cyan, form I protomer 2; pink, form I protomer 3; purple, formII extrusion protomer). (b) The position of side chains of residues D409, D410 and K935 within the proton relay network (green, form I protomer 1; cyan, form I protomer 2; pink, form I protomer 3; purple, formII extrusion protomer).

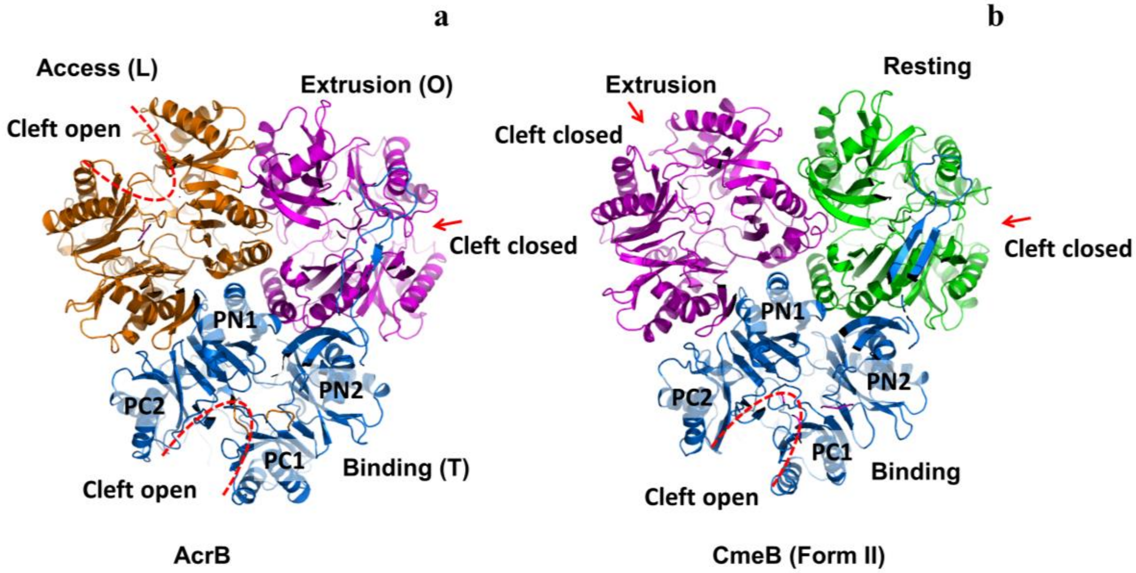

Supplementary Figure 6. Comparison of the crystal structures of AcrB and CmeB (form II). (a) Top view of the AcrB structure. The three protomers of AcrB are colored (brown, access (L) protomer; blue, binding (T) protomer; magenta, extrusion (O) protomer). (b) Top view of the form II structure of CmeB. The three protomers of the form II structure are colored (green, resting protomer; blue, binding protomer; magenta, extrusion protomer).

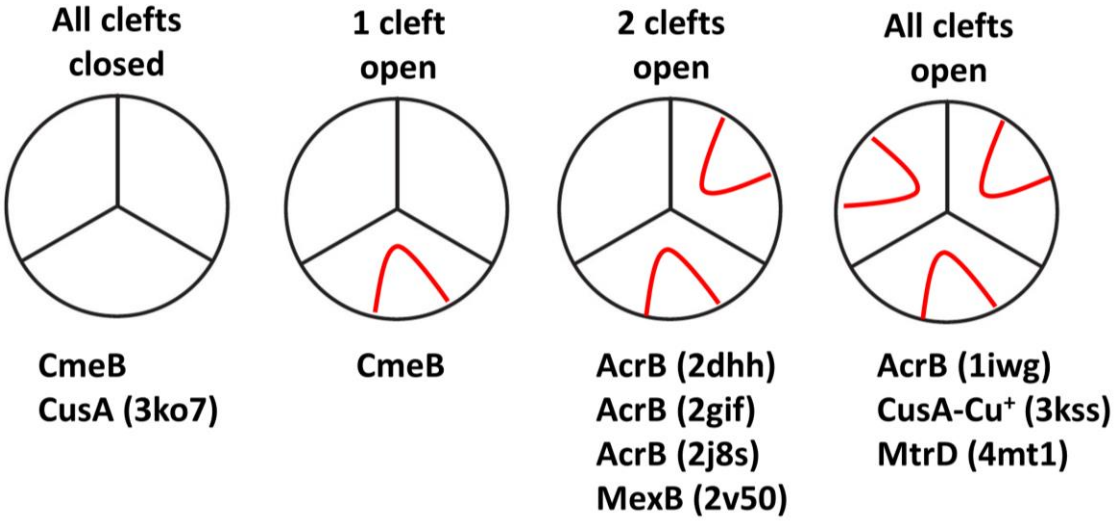

Supplementary Figure 7. Different conformations of the periplasmic domains of RND efflux pumps. The structures suggest that the periplasmic clefts formed by subdomains PC1 and PC2 could open or closed independently.

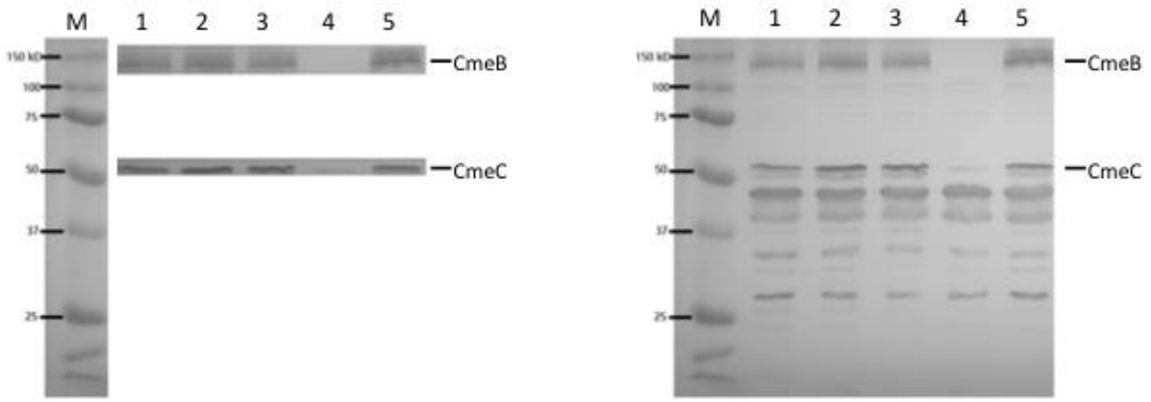

1. 81-176
2. 81-176ΔcmeABC::cat16s::cmeABC (D409A)
3. 81-176ΔcmeABC::cat16s::cmeABC (D410A)
4. 81-176ΔcmeABC::cat
5. 81-176ΔcmeABC::cat16s::cmeABC

Supplementary Figure 8. Expression level of the CmeB pumps. An immunoblot against CmeB of crude extracts from 50 μg dry cells of *C. jejuni* 81-176 strain expressing the CmeB wild-type and mutant (D409A and D410A) pumps are shown (left, cropped; right, uncropped).

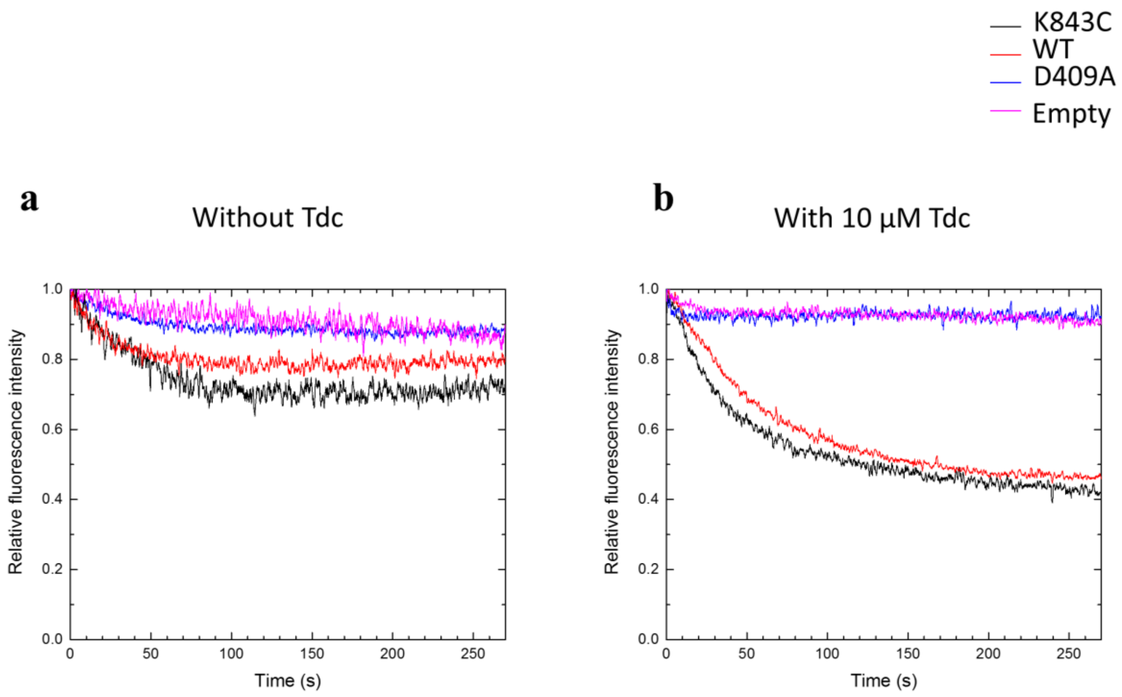

Supplementary Figure 9. Transport assay of reconstituted CmeB with intravesicular and extravesicular pHs at 7.5 and 6.5, respectively. (a) The experiments were done in the absence of Tdc. The decrease in fluorescence signal of pyranine mediated by proteoliposomes of wild-type CmeB and the K843C mutant indicate the translocation of protons across the membrane. The stopped-flow traces are the cumulative average of four successive recordings (wild-type CmeB, red curve; K843C mutant, black curve; D409A mutant, blue curve; liposomes without protein, magenta curve). (b) The experiments were done in the presence of 10 μM Tdc. The decrease in fluorescence signal of pyranine mediated by proteoliposomes of wild-type CmeB and the K843C mutant indicate the translocation of protons across the membrane. The stopped-flow traces are the cumulative average of four successive recordings (wild-type CmeB, red curve; K843C mutant, black curve; D409A mutant, blue curve; liposomes without protein, magenta curve).

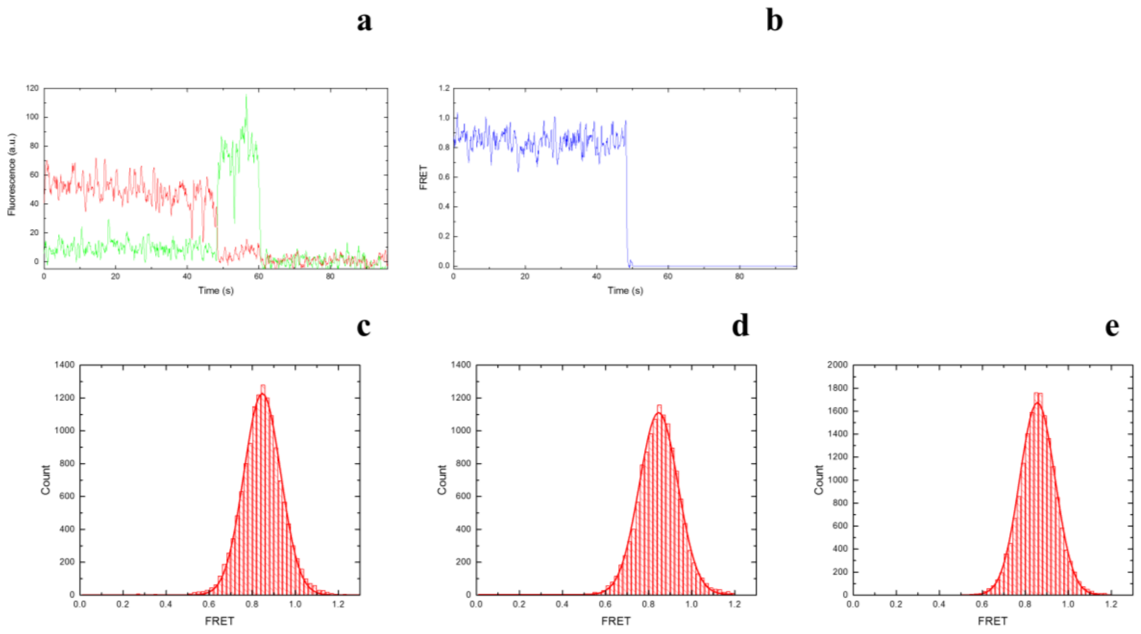

Supplementary Figure 10. Dynamics in the apo and substrate bound K781C mutant pump. (a) Representative traces of K781C single-molecule dynamics with donor (green) and acceptor (red) fluorescence in the absence of Tdc. A 5-frame moving average filter was applied to the traces to reduce noise (b) The constant FRET efficiency before photobleaching, showing the lack of major conformational changes in funnel domain. The FRET state distributions (c) in the absence of Tdc, (d) in the presence of 1 μM Tdc and (e) in the presence of 10 μM Tdc. The histograms were fitted with a single Gaussian function.

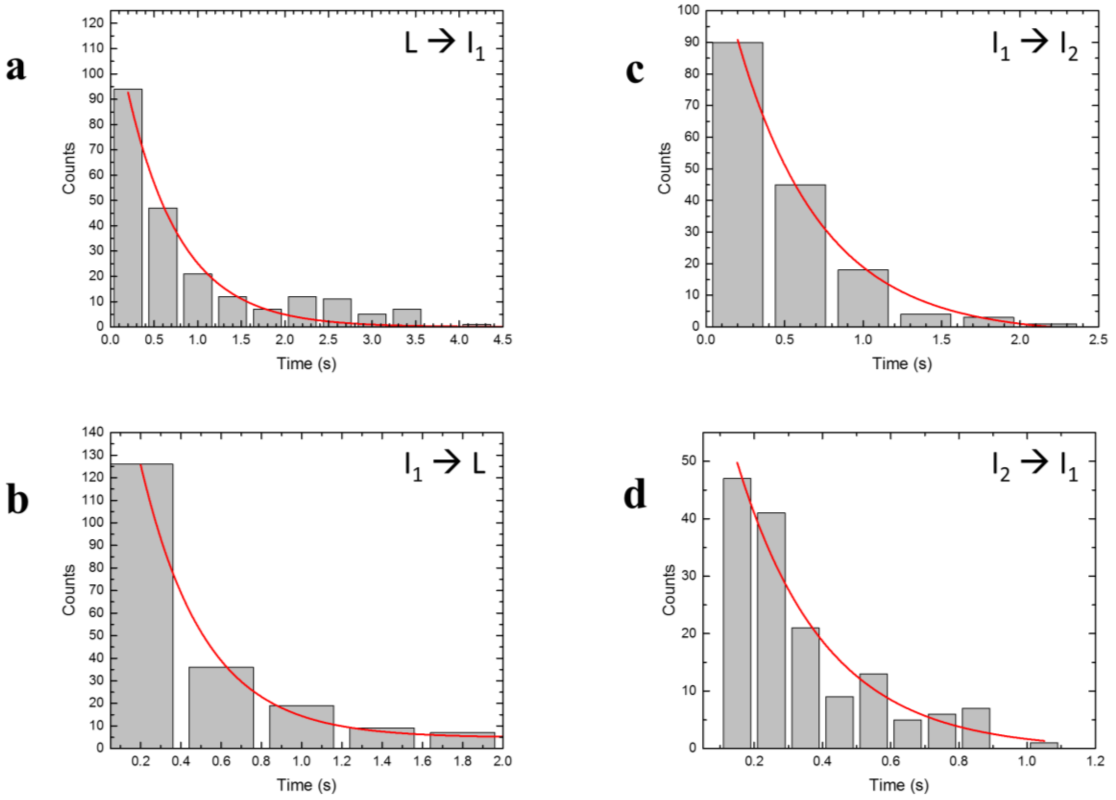

Supplementary Figure 11. Distribution of dwell times for CmeB (K843C) transitions in the absence of ligand. The exponential lifetime can be obtained by fitting the data with a single-exponential function, resulting in (a) $\tau_{L \rightarrow I1}$ = 0.61 s ($k_{L \rightarrow I1}$ = 1.64 s^{-1}), (b) $\tau_{I1 \rightarrow L}$ = 0.31 s ($k_{I1 \rightarrow L}$ = 3.22 s^{-1}), (c) $\tau_{I1 \rightarrow I2}$ = 0.54 s ($k_{I1 \rightarrow I2}$ = 1.85 s^{-1}) and (d) $\tau_{I2 \rightarrow I1}$ = 0.26 s ($k_{I2 \rightarrow I1}$ = 3.84 s^{-1}).

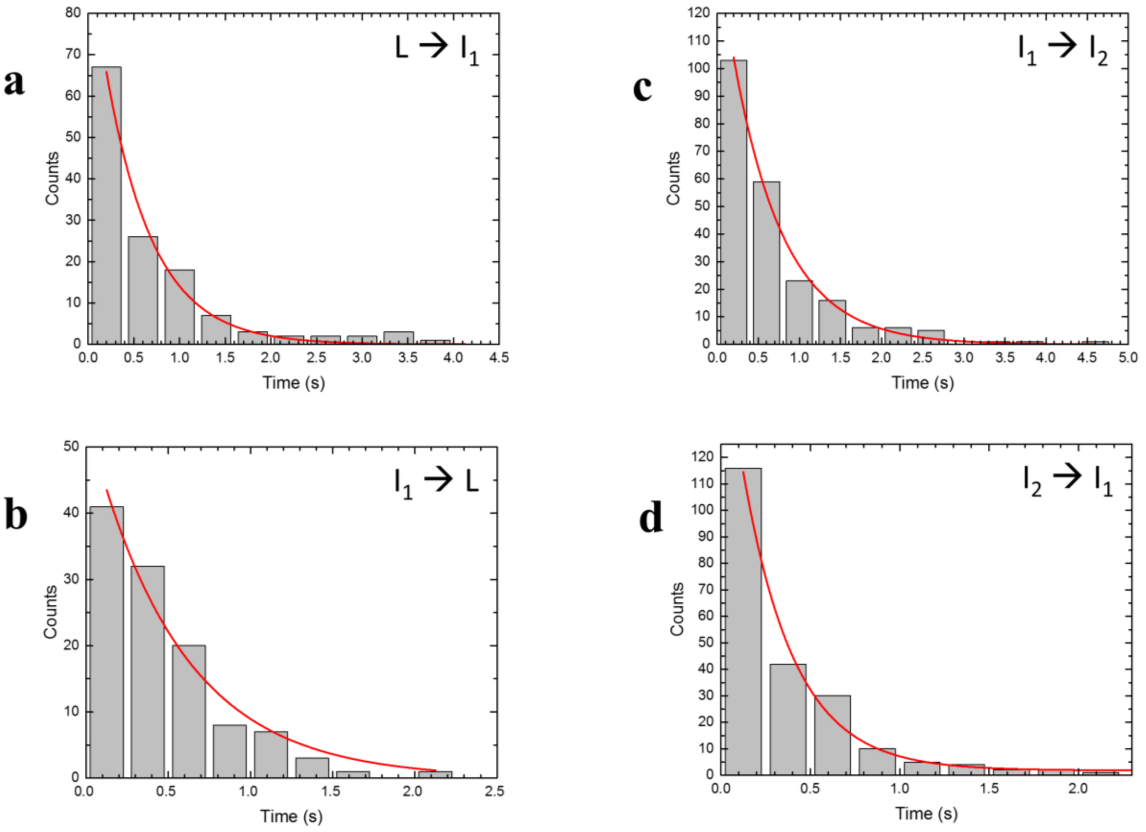

Supplementary Figure 12. Distribution of dwell times for CmeB (K843C) transitions in the presence of 1 μM Tdc. The exponential lifetime can be obtained by fitting the data with a single-exponential function, resulting in (a) $\tau_{L\rightarrow I1}$ = 0.52 s ($k_{L\rightarrow I1}$ = 1.92 s^{-1}), (b) $\tau_{I1\rightarrow L}$ = 0.55 s ($k_{I1\rightarrow L}$ = 1.82 s^{-1}), (c) $\tau_{I1\rightarrow I2}$ = 0.62 s ($k_{I1\rightarrow I2}$ = 1.61 s^{-1}) and (d) $\tau_{I2\rightarrow I1}$ = 0.29 s ($k_{I2\rightarrow I1}$ = 3.45 s^{-1}).

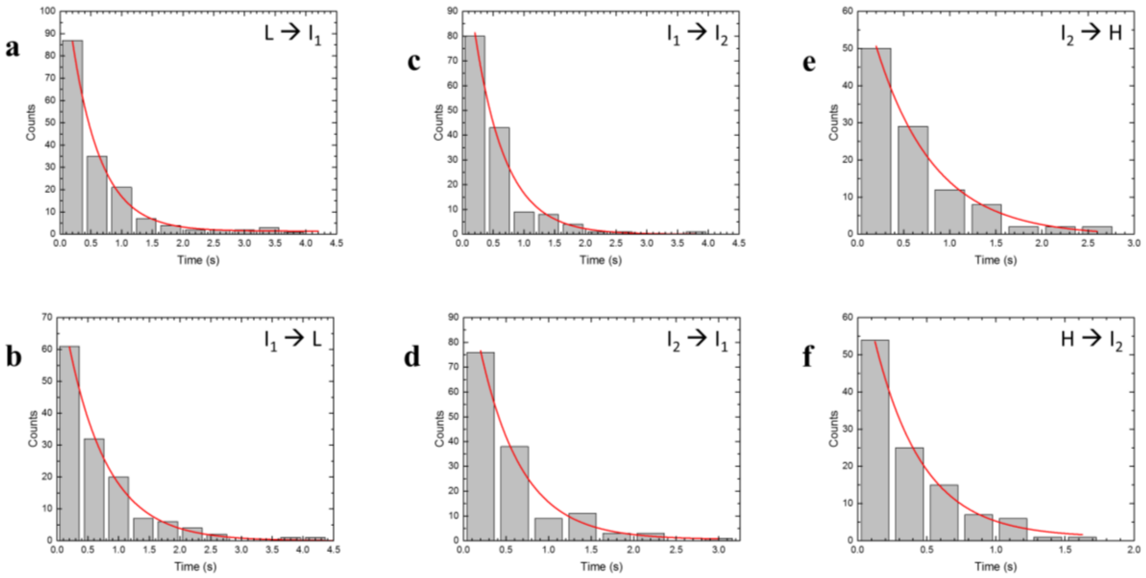

Supplementary Figure 13. Distribution of dwell times for CmeB (K843C) transitions in the presence of 10 μM Tdc. The exponential lifetime can be obtained by fitting the data with a single-exponential function, resulting in (a) $\tau_{L \rightarrow I1}$ = 0.47 s ($k_{L \rightarrow I1}$ = 2.13 s^{-1}), (b) $\tau_{I1 \rightarrow L}$ = 0.65 s ($k_{I1 \rightarrow L}$ = 1.54 s^{-1}), (c) $\tau_{I1 \rightarrow I2}$ = 0.50 s ($k_{I1 \rightarrow I2}$ = 2.00 s^{-1}), (d) $\tau_{I2 \rightarrow I1}$ = 0.49 s ($k_{I2 \rightarrow I1}$ = 2.04 s^{-1}), (e) $\tau_{I2 \rightarrow H}$ = 0.63 s ($k_{I2 \rightarrow H}$ = 1.59 s^{-1}) and (f) $\tau_{H \rightarrow I2}$ = 0.35 s ($k_{H \rightarrow I2}$ = 2.86 s^{-1}).

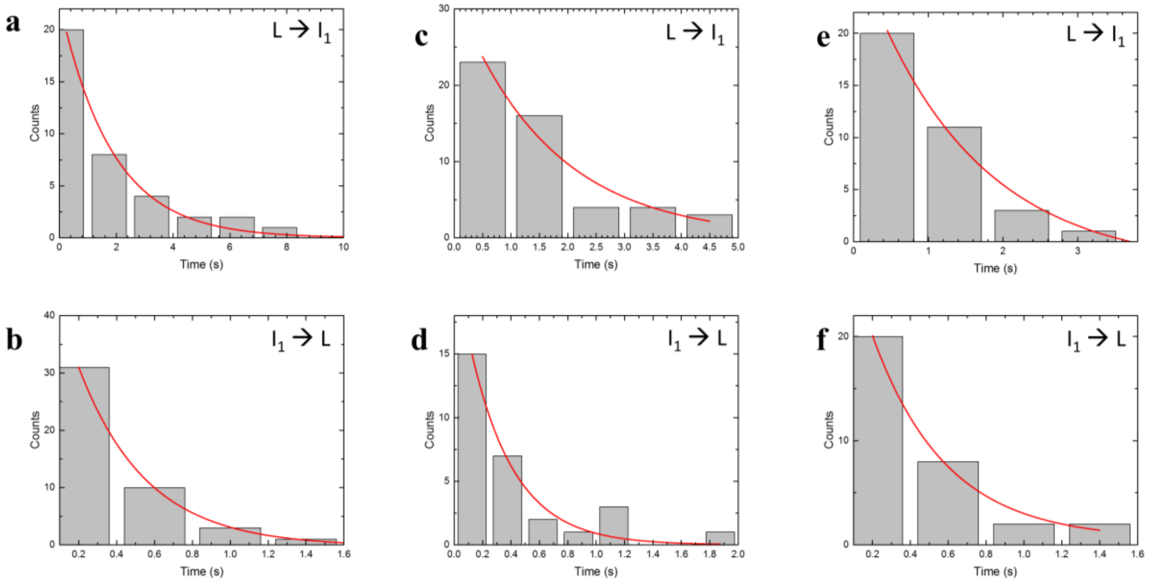

Supplementary Figure 14. Distribution of dwell times for CmeB (K843C-D409A) transitions. The exponential lifetime can be obtained by fitting the data with a single-exponential function. In the absence of ligand, the resulting dwell times are (a) $\tau_{L\rightarrow I1}$ = 1.85 s ($k_{L\rightarrow I1}$ = 0.54 s^{-1}) and (b) $\tau_{I1\rightarrow L}$ = 0.35 s ($k_{I1\rightarrow L}$ = 2.86 s^{-1}). In the presence of 1 μM Tdc, the resulting dwell times are (c) $\tau_{L\rightarrow I1}$ = 1.68 s ($k_{L\rightarrow I1}$ = 0.60 s^{-1}) and (d) $\tau_{I1\rightarrow L}$ = 0.31 s ($k_{I1\rightarrow L}$ = 3.22 s^{-1}). In the presence of 10 μM Tdc, the resulting dwell times are (e) $\tau_{L\rightarrow I1}$ = 1.47 s ($k_{L\rightarrow I1}$ = 0.68 s^{-1}) and (f) $\tau_{I1\rightarrow L}$ = 0.39 s ($k_{I1\rightarrow L}$ = 2.56 s^{-1}).

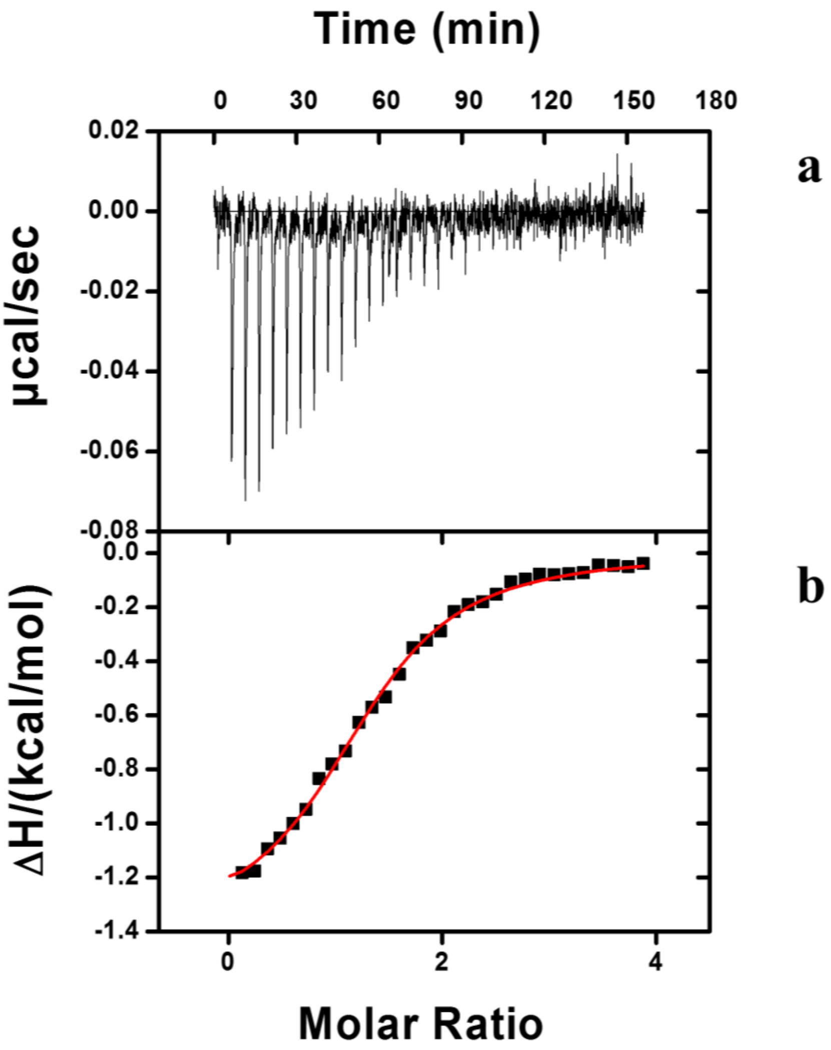

Supplementary Figure 15. Representative isothermal titration calorimetry for the binding of taurodeoxycholate to CmeB. (a) Each peak corresponds to the injection of 10 μl of 250 μM taurodeoxycholate in buffer containing 20 mM Na-HEPES pH 7.5 and 0.03% DDM into the reaction containing 20 μM monomeric CmeB in the same buffer. (b) Cumulative heat of reaction is displayed as a function of the injection number. The solid line is the least-square fit to the experimental data, giving a K_D of 3.26 ± 0.21 μM.

Supplementary Table 1. Difference in structures between CmeB (form I) and AcrB.

			PN1+PC2				PN2+PC1				TM1-6				TM7-12	
			AcrB				AcrB				AcrB				AcrB	
		L	T	O		L	T	O		L	T	O		L	T	O
CmeB	A	0.7	0.6	0.7		1.0	1.7	0.9		1.4	1.3	0.9		1.0	1.1	0.8
	B	0.8	0.7	0.8		1.1	1.8	0.9		1.4	1.3	0.9		1.0	1.2	0.8
	C	0.7	0.6	0.7		1.1	1.7	1.0		1.4	1.3	0.9		1.0	1.0	0.8

Difference in structures of repeats in the periplasmic and transmembrane domains of form I CmeB, in chain A, B, C vs. AcrB in the L, T, and O states. The porter domain in each CmeB protomer consists of two repeats, referred to as PN1+PC2 and PC1+PN2, (PN1: residues 42 to 50, 77 to 84, 87 to 95 and 127 to 132; PC2: residues 674 to 680, 708 to 712, 818 to 824, 851 to 855; PC1: residues 568 to 576, 603 to 610, 621 to 629 and 57 to 662; PN2: residues 138 to 144, 174 to 178, 288 to 294, 324 to 328). The differences between two structures or conformational states are quantified in terms of the root-mean-squared difference (RMSD) between them, after least-squares fitting; all values are given in Å. This analysis shows that the structures of PN1+PC2 repeat of CmeB and AcrB are very similar in all the states. However, conformations of PC1+PN2, TM1-6 and TM7-12 vary significantly and all conformation of form I CemB protomers are close to O protomer in AcrB.

Supplementary Table 2. Difference in structures between CmeB (form II) and AcrB.

			PN1+PC2				PN2+PC1				TM1+TM3-6				TM7+TM9-12	
			AcrB				AcrB				AcrB				AcrB	
		L	T	O		L	T	O		L	T	O		L	T	O
CmeB	E	0.8	0.7	0.9		1.0	1.7	0.9		1.3	1.3	0.9		1.0	1.0	0.8
	B	0.8	0.8	0.9		2.3	0.9	2.1		1.2	0.9	1.4		0.9	0.9	1.3
	R	0.8	0.7	0.8		1.0	1.7	0.9		1.3	1.3	0.9		1.0	1.1	0.8

Difference in structures of form II CmeB, in extrusion (E), binding (B), resting (R) vs. AcrB in the L, T, and O states. This analysis shows that the structures of PN1+PC2 repeat of CmeB and AcrB are very similar in all the states. However, structural alignments of PC1+PN2, TM1-6 and TM7-12 indicate B protomer of CmeB is closed to T state, while E and R protomers are more closed to O state of AcrB.

Supplementary Table 3. Difference between the form I and form II structures of CmeB.

		PN1+PC2			PN2+PC1			TM1+TM3-6			TM7+TM9-12		
		Form I			Form I			Form I			Form I		
		A	B	C	A	B	C	A	B	C	A	B	C
Form II	E	0.4	0.4	0.4	0.5	0.5	0.5	0.4	0.4	0.4	0.4	0.4	0.4
	B	0.7	0.7	0.7	1.7	1.7	1.6	1.5	1.5	1.5	1.0	1.0	1.0
	R	0.4	0.5	0.4	0.5	0.5	0.5	0.5	0.5	0.5	0.4	0.5	0.4

Difference in structures of the repeats in the periplasmic and transmembrane domains of form I CmeB, in chain A, B, C vs. form II CmeB in extrusion (E), binding (B), resting(R) states.

Supplementary Table 4. MICs of taurocholate, taurodeoxycholate, and rifampin for different CmeB variants in the *C. jejuni* 81-176.

C. jejuni strain	MIC (mg liter^{-1})		
	Taurocholate	Taurodeoxycholate	Rifampin
81-176	>25000	>5120	128
81-176 *ΔcmeABC::cat*	781	80	0.5
81-176 *ΔcmeABC::cat 16S::cmeABC*	>25000	>5120	128
81-176 *ΔcmeABC::cat 16S::cmeABC (3C-3S)*	>25000	>5120	128
81-176 *ΔcmeABC::cat 16S::cmeABC (3C-3S, K781C)*	>25000	>5120	128
81-176 *ΔcmeABC::cat 16S::cmeABC (3C-3S, K843C)*	>25000	>5120	128
81-176 *ΔcmeABC::cat 16S::cmeABC (3C-3S, D409A)*	781	80	0.5
81-176 *ΔcmeABC::cat 16S::cmeABC (3C-3S, D410A)*	1562	80	2

Supplementary Table 5. Thermodynamic parameters of CmeB binding reactions.

	K_D (μM)	ΔH	ΔS
TDC	3.26 ± 0.21	-1392.0 ± 26.62	20.4
TDC in 2mM COT	2.79 ± 0.20	-565.4.5 ± 5.87	23.5

Supplementary Notes

In vivo antimicrobial susceptibility assay

We used the *C. jejuni 81-176 ΔcmeABC::cat* null mutant strain, which lacks the *cmeABC* genes. We inserted the *cmeABC* operon that includes *cmeABC*, *cmeR* and the intergenic region between *cmeR* and *cmeA* into the 16S region of the 81-176 genomic DNA. This approach allowed us to ensure that the expression of *cmeB* was driven from a single copy of gene in the genomes with native regulator, operator and promoter. The expression level of *cmeABC* was determined using the anti-CmeB and anti-CmeC antibodies. Western analysis suggested that the expression level of wild-type CmeB, D409A and D410 mutant transporters were more or less the same (Supplementary Figure 8).

We then tested the susceptibility of *C. jejuni* cells carrying wild-type CmeB or its isogenic mutant (D409A or D410A) to taurocholate, taurodeoxycholate and rifampin. These three antimicrobials are the known substrates of the CmeABC efflux pump[9]. We found that *C. jejuni* cells expressing the D409A or D410A mutant were >64-fold less sensitive to taurodeoxycholate when compared with *C. jejuni* cells carrying the wild-type CmeB pump (Supplementary Table 4). In addition, cells producing the D409A and D410A variants were >32-fold and >16-fold, respectively, less resistance to taurocholate when compared with cells expressing the wild-type pump. We also found that the minimum inhibitory concentrations (MICs) of *C. jejuni* cells producing D409A and D410A to rifampin were at least 256 and 64 times, respectively, lower than those of *C. jejuni* cells carrying wild-type CmeB (Supplementary Table 4). These data show that both D409 and D410 residues are critical for the function of the CmeB pump.

Binding of taurodeoxycholate by CmeB

We used isothermal titration calorimetry (ITC) to determine the binding affinity of taurodeoxycholate (Tdc) for the CmeB multidrug efflux pump. The data indicate that the dissociation constant, K_D, for Tdc binding is 3.26 ± 0.21 μM (Supplementary Figure 15 and Supplementary Table 5), confirming the purified CmeB protein is capable of recognizing this bile acid.

As cyclooctatetraene was needed in our FRET experiments to reduce the lifetime of dark states, we also determined the binding affinity of Tdc for CmeB in the presence of 2 mM cyclooctatetraene. ITC data indicated that the K_D for Tdc binding is 2.79 ± 0.20 μM, suggesting that the presence of cyclooctatetraene does not affect the binding affinity of Tdc (Supplementary Table 5).

***In vitro* proton translocation across the CmeB proteoilposomes**

We next examined if protons can translocate across the lipid bilayer of the CmeB proteoliposomes. The purified CmeB protein was reconstituted into liposomes containing the fluorescence proton-specific probe pyranine[43] in the intra-vesicular space, where the pH was adjusted to 7.5. When added into buffer solution containing 20 mM Na-HEPES (pH 6.5), we detected a significant quenching of the fluorescence signal in proteoliposomes possessing wild-type CmeB compared with those liposomes without the pump.

We then investigated whether protons can be transferred in the presence of 10 μM Tdc. When Tdc was added into the extravesicular medium of the CmeB proteoliposomes,

we detected a much stronger quenching of the fluorescence signal compared with that in the absence of Tdc. In addition, we did not detected any quenching signal for protein free liposomes in the presence of 10 μM Tdc, indicating that the presence of Tdc does not affect the integrity of the liposomes. Our data suggest that the process of proton translocation is much more effective in the presence of CmeB substrates.

The crystal structure of CmeB suggests that the charged residue D409 in the transmembrane domain may be important for proton translocation. When reconstituted into liposomes, the mutant transporter D409A did not transfer protons into the intravesicular space (Supplementary Figure 9), thus confirming the importance of residue D409 for the function of the pump.
